# Genome-Wide Scan for Methylation Profiles in Keloids

**DOI:** 10.1155/2015/943176

**Published:** 2015-05-14

**Authors:** Lamont R. Jones, William Young, George Divine, Indrani Datta, Kang Mei Chen, David Ozog, Maria J. Worsham

**Affiliations:** ^1^Department of Otolaryngology-Head and Neck Surgery, Henry Ford Hospital, Detroit, MI 48202, USA; ^2^Proliance Eastside ENT, Kirkland, WA 98033, USA; ^3^Department of Public Health Sciences, Henry Ford Health System, Detroit, MI 48202, USA; ^4^Center for Bioinformatics, Henry Ford Health System, Detroit, MI 48202, USA; ^5^Department of Dermatology, Henry Ford Hospital, Detroit, MI 48202, USA

## Abstract

Keloids are benign fibroproliferative tumors of the skin which commonly occur after injury mainly in darker skinned patients. Medical treatment is fraught with high recurrence rates mainly because of an incomplete understanding of the biological mechanisms that lead to keloids. The purpose of this project was to examine keloid pathogenesis from the epigenome perspective of DNA methylation. Genome-wide profiling used the Infinium HumanMethylation450 BeadChip to interrogate DNA from 6 fresh keloid and 6 normal skin samples from 12 anonymous donors. A 3-tiered approach was used to call out genes most differentially methylated between keloid and normal. When compared to normal, of the 685 differentially methylated CpGs at Tier 3, 510 were hypomethylated and 175 were hypermethylated with 190 CpGs in promoter and 495 in nonpromoter regions. The 190 promoter region CpGs corresponded to 152 genes: 96 (63%) were hypomethylated and 56 (37%) hypermethylated. This exploratory genome-wide scan of the keloid methylome highlights a predominance of hypomethylated genomic landscapes, favoring nonpromoter regions. DNA methylation, as an additional mechanism for gene regulation in keloid pathogenesis, holds potential for novel treatments that reverse deleterious epigenetic changes. As an alternative mechanism for regulating genes, epigenetics may explain why gene mutations alone do not provide definitive mechanisms for keloid formation.

## 1. Introduction

Keloids are benign fibroproliferative tumors that are unique to humans [1]. The exact mechanism of keloid formation is unknown. There is up to a 20% incidence in Blacks, Asians, and Hispanics [2–7]. Dysregulations of genes important in apoptosis, extracellular matrix formation, and immunity have been described in the pathogenesis of keloids [8, 9]. However, no clear-cut genetic alterations or involvement have emerged for keloids. Clinical outcomes for the treatment of keloids are disappointing. Surgical excision of keloids has a 50–100% recurrence [10]. Furthermore, the recurrence rate of surgical excision combined with adjuvant therapy, such as steroid injection, silicone therapy, pressure therapy, radiotherapy, and anticancer drugs, remains high with up to 50% reported in the literature [3]. The failure of adjuvant therapies, despite being based on current clinical, histological, or molecular observations, underscores the heterogeneity of keloid formation and the need for insight into the molecular pathogenesis of keloids to account for its complexity.

Keloid molecular studies have focused primarily on genetic mechanisms. These approaches have yielded some tangible results, albeit with large gaps in our understanding of keloid pathogenesis [11]. Epigenetics is the study of gene alteration that occurs without altering the DNA sequence. For example, DNA hypermethylation is used to turn off genes in normal human development, such as imprinting and X chromosomal inactivation [12–14]. Aberrant methylation, the addition of a methyl group (CH_3_) at cytosine bases of DNA (hypermethylation), or removal (hypomethylation) can lead to genomic instability and tumorigenesis [15]. Hypermethylation in the cytosine-phosphodiester bond-guanine (CpG) islands of a gene's promoter region can result in gene inactivation, whereas hypomethylation can cause gene activation [16–18]. Recent evidence suggests that DNA methylation changes may underlie numerous complex traits and diseases [19–21]. As an alternative mechanism for regulating genes, epigenetics may explain why gene mutations alone do not provide definitive mechanisms for keloid formation.

Historically, the molecular pathogenesis of disease has been teased out one gene at a time. The development of several new high-throughput methods for the analysis of DNA, mRNA, and proteins within a cell has permitted a more detailed molecular characterization of the cancer genome. Given the tumor-like behavior of keloids, it is plausible that similar techniques could be applied to the study of keloid pathogenesis. Studies have shown downregulation of apoptotic genes in keloid tissue, including those that both promote and inhibit apoptosis [22]. Furthermore, immunohistochemistry data indicate upregulation of tumor suppressor genes, such as p53, and protooncogenes, such as bcl2 [16]. However, these methods have not adequately addressed the complexity of the disease. A combination of genomic, epigenomic, and environmental factors more likely underscores the pathogenesis of complex diseases such as keloids.

The objective of this study was to gain insight into keloid pathogenesis from the epigenome perspective of DNA methylation. This study used a global discovery strategy to characterize the keloid methylome and identify keloid-specific methylated markers to aid in the understanding of the disease.

## 2. Materials and Methods

Discarded anonymous human keloid and normal skin tissue was obtained from patients via a protocol approved by the institutional review board. Six fresh keloid and 6 normal skin samples from the head and neck area from 12 anonymous donors were obtained for the study cohort. Normal skin was chosen as a control because keloids form from normal skin. Genome-wide profiling was done using the Infinium HumanMethylation450 BeadChip platform.

### 2.1. Distribution and Classification of the 450,000 Cytosine Sites in the Human Genome

The Infinium HumanMethylation450 BeadChip platform provides coverage of 99% of Reference Sequence genes. The Reference Sequence collection aims to provide a comprehensive, integrated, nonredundant, well-annotated set of sequences, including genomic DNA, transcripts, and proteins [23]. The 450K DNA methylation array includes 485,764 cytosine positions in the human genome. From these cytosine sites, 482,421 positions (99.3%) are CpG dinucleotides, while 3,343 sites (0.7%) correspond to non-CpG targets. The interrogated CpG sites are distributed among all human 22 autosomal and 1 sex chromosome pairs [23]. From the functional genome distribution standpoint, 200,339 CpGs (41%) are located in proximal promoters and became the focus of analyses for this study.

### 2.2. Processing Samples for the Infinium HumanMethylation450

The Infinium HumanMethylation450 assays were performed at the Applied Genomics Technology Center, Wayne State University, Detroit, MI. DNA was extracted according to the manufacturer's protocol (Qiagen Inc., Chatsworth, CA). Following DNA quality checks of original DNA quality, quantification, and bisulfite conversion, 4 *µ*L of bisulfite-converted DNA was used for hybridization according to Illumina Infinium methylation protocols. Data were normalized using the Controls Normalization method (Illumina, San Diego, CA). The methylation score for each CpG was represented as a beta (*β*) value according to the fluorescent intensity ratio. Every *β*-value in the Infinium HumanMethylated450 BeadChip platform was accompanied by a detection *P* value. *β*-values may take any value between 0 (nonmethylated) and 1 (completely methylated) and were determined using the Genome Studio V2009.2, Methylation Module Version 1.5.5., Version 3.2 (Illumina, San Diego, CA). Probes were discarded if this detection *P* value was more than 0.05. The only corrections that were made to the data were background subtraction and normalization.

The resulting *β*-values were exported into Microsoft Excel, JMP, and SAS (SAS Institute, Cary, NC) for data analysis. All genome-wide comparisons were corrected for multiple comparisons using the method of Benjamini and Hochberg [24].

### 2.3. Data Analysis

To identify differentially methylated keloid-specific genes, a three-tiered approach was developed [25]. Our goal for a 3-Tier system was to provide a framework to increase statistical rigor in the detection of biologically relevant methylation markers. The latter achieves two outcomes: (1) exclusion of CpGs likely increases the risk of false discoveries and (2) serves as a strategy to reduce the number of genes/CpGs for confirmation especially for study cohorts with DNA and RNA sources challenged by formalin-fixation or meager amounts of tissue availability for molecular characterization [25]. In Tier 1, we computed adjusted false discovery rate values for all CpGs and required this to be 0.05 or lower. In Tier 2, in order to heuristically move from Tier 1, we filtered the Tier 1 CpGs to include only those with a twofold change (ratio ≥ 2.0 or ≤ 0.5). Tier 3 added another CpG filter to include only those with an absolute difference between the mean *β* of ≥0.2. A CpG was classified as associated with a promoter region if the Illumina annotation for any of the associated gene isoforms was designated as translation start site (TSS) 200, TSS1500, 5′untranslated region (UTR), or 1st exon. Nonpromoter regions included gene body, 3′UTR, and others (intergenic). The addition of Tier 2 and 3 criteria was intended to downsize the number of CpGs/genes whose differential methylation likely represented the most robust of biologically significance. This study focused on promoter region CpGs; therefore, only Tier 3 CpGs were correlated for associated genes.

## 3. Results

**Figure 1:**
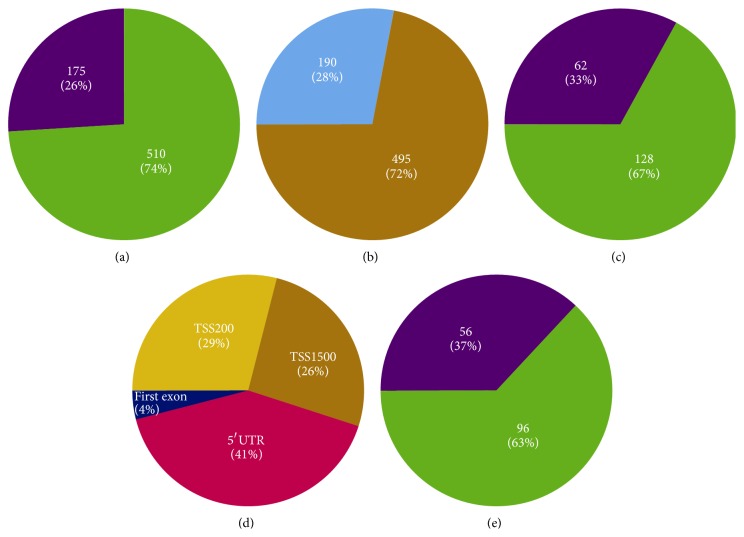
(a) Methylation status of the 685 differentially methylated CpGs (cytosine-phosphodiester bond-guanine) at Tier 3 (26% hypermethylated, 74% hypomethylated). (b) 685-CpG distributions in promoter versus nonpromoter (28% promoter, 72% nonpromoter). (c) Methylation status of 190 promoter CpGs (33% hypermethylated, 67% hypomethylated). (d) 190-CpG distribution in promoter regions (41% 5′UTR, 29% TSS200, 26% TSS1500, and 4% 1st exon). (e) Methylation status of 152 genes associated with the 190 CpGs (37% hypermethylated, 63% hypomethylated).

With respect to this sample set, >99.7% of the 485,577 Illumina probes had detection *P* values under 0.05. Of the 485,577 cytosine positions, when compared to normal, Tier 1 yielded 29,722 CpGs (24,280 hyper- and 5442 hypomethylated), Tier 2 yielded 1,534 differentially methylated CpGs (551 hyper- and 983 hypomethylated), and Tier 3 yielded 685 differentially methylated CpGs. Of the CpGs at Tier 3, 175 were hypermethylated and 510 were hypomethylated with 190 CpGs (28%) in promoter and 495 (72%) in nonpromoter regions. The predominant location of differentially methylated CpGs in nonpromoter regions as compared to promoter regions was also observed for CpGs at Tier 1 and Tier 2 levels (Figure 1, Table 1).

Of the 190 promoter region CpGs, 128 were hypomethylated and 62 were hypermethylated with a functional distribution of 29% in TSS200, 26% in TSS1500, 41% in 5′UTR, and 4% in the first exonic region. The 190 Tier 3 CpGs mapped to 197 CpG/gene combinations, which involve 152 genes. The 152 genes include 96 (63%) hypomethylated and 56 (37%) hypermethylated (Figure 1, Table 1, and Supplementary Table 1 in Supplementary Material available online at http://dx.doi.org/10.1155/2015/943176).

## 4. Discussion

Keloids share many similarities with the process of tumorigenesis. Morphologically, keloid pathogenesis is characterized by overgrowth. Hyperplasia is a key trait and almost always is a precursor in tumorigenesis along a malignancy continuum. Analogies can be drawn between keloids and malignancies in terms of their biological behavior, such as rapid proliferation and aberrant genetic profiles, for example, the upregulation of tumor suppressor genes such as p53 and protooncogenes such as bcl2 [22]. Keloids thus can be described as being pseudomalignant in nature. Studies support methylated genes not only as biomarkers of benign tumors, such as sinonasal [26] and respiratory papillomas [27, 28], but also along a continuum from normal to benign to malignant [29, 30]. We hypothesized that the analogy of an epigenetic milieu might extend to keloids as well.

Of the two types of aberrant methylation patterns present in cancer cells [31, 32], one is gene-specific hypermethylation, where CpG islands in the promoter regions of genes acquire increased methylation, generally leading to reduced expression of the gene. The other is genome-wide hypomethylation, a large percentage of which occurs in repetitive DNA elements such aslong interspersed nuclear element (LINE) and short interspersed nuclear element (SINE, called Alu in primates) in a variety of cancer cell lines and primary tumor samples [31, 33]. In malignancy, aberrant gene-specific methylation is often increased while global methylation is often aberrantly reduced [31, 32]. While DNA methylation is generally associated with transcriptional silencing, the effects of reduced global methylation or genome-wide hypomethylation can lead to chromosomal instability and an increase in the frequency of DNA strand breaks [34].

From a genome-wide perspective, the emergence of a more hypomethylated keloid gene promoter landscape shows similarity with nonmalignant as well as normal developmental processes such as successful placentation [35]. A hypomethylated keloid genomic landscape contrasts with malignant tumor genomes, such as in breast cancer [36], which shows more hypermethylated than hypomethylated genes. Promoter DNA hypomethylation often results in gene activation. The progression of scars in normal skin after injury to a keloid pseudo tumor state may be explained by gene silencing from hypomethylation. Our study suggests that benign tumors adopt hypomethylation as a mechanism for gene activation rather than gene silencing to cross thresholds into more malignant states. The latter was illustrated for peptidase activity, shown to be hypomethylated across cancers and required for the tumor cells to break through the extracellular matrix and basement membrane barriers to become invasive, and thus its predicted upregulation via hypomethylation to promote metastasis [37]. Other hypomethylated concepts such as epidermal and keratinocyte development and differentiation have been linked to worse survival prognosis and increased local invasiveness [38]. The overall global hypomethylation status of the differentially methylated keloid genes, despite a small discovery sample set, is a novel contrast with cancers such as breast and head and neck which are predominantly hypermethylated genomes.

Another observation is the predominant location of differentially methylated CpGs in nonpromoter regions such as gene body, 3′UTR, and intergenic as compared to promoter regions at all three tier levels. DNA methylation at intergenic sequences may play roles in regulating gene expression and/or chromosome compaction during cell division and meiosis [39]. Recent genome-wide approaches indicate that DNA methylation can frequently occur in regions outside of proximal promoters, including intergenic sequences [40, 41]. Evolutionarily conserved nonprotein coding regions that were also tissue differentially methylated regions were shown to have locations of up to 100 kb from the nearest annotated gene, consistent with potential long-range regulatory elements such as silencers or enhancers [42].

Of the differentially methylated genes in our exploratory methylome scan, more than a third were hypermethylated. Several human therapeutic intervention trials are underway to reverse deleterious hypermethylated epigenetic changes. Examples include epigenetic therapeutic trials to treat T-cell lymphoma based on reactivation of tumor suppressor genes [43] and similar trials to prevent colorectal cancer by inhibiting the enzyme responsible for DNA methylation [44]. Such therapies have shown promise in halting tumor growth by reactivation of the tumor suppressor gene or by blocking progression of precancerous epigenetic lesions.

Epigenomic biomarkers are also becoming far more practical than genomic biomarkers. Our group has shown that promoter hypermethylation is amenable to polymerase chain reaction- (PCR-) based methylation assays using whole genomic DNA from fresh/frozen tissue and cell lines, as well as formalin-fixed paraffin tissue DNA [26–28]. Methylation of CpG islands may serve as a relatively simple “yes-no” signal for the presence of tumor, and potentially the pathogenesis of keloid tissue, when examined for under optimal assay conditions by sensitive PCR techniques [26–29]. Additionally, aberrant promoter hypermethylation always occurs in virtually the same location within an affected gene, allowing a single PCR primer to be applicable to all patients for examination of the methylation status of a specific gene [32]. This sharply contrasts with genomic biomarkers such as DNA mutations in genes, such as p53 or mitochondrial genes [29], which often involve myriad different base changes at many locations within the gene even in cancers of the same histologic types. Classification based on promoter methylation profiling may well be a more promising approach than expression profiling since these DNA-based techniques are not subject to the problems of tissue preservation and the potential pitfalls of tissue heterogeneity. Aberrant DNA methylation, as a stable biomarker of keloid pathogenesis or a potential target, could be easily detected by PCR-based methods. Furthermore, methylation profiles may represent an early marker for the initiation, development, and/or progression of keloid pathogenesis.

Of the differentially methylated genes in our exploratory methylome scan, more than a third were hypermethylated. The potential reversibility of DNA methylation patterns may serve as a target for therapy or as a marker for adjuvant therapy or as an adjunct to more conventional treatments such as surgical excision and steroid injections. Several human therapeutic intervention trials are underway to reverse deleterious hypermethylated epigenetic changes. Examples include epigenetic therapeutic trials to treat T-cell lymphoma based on reactivation of tumor suppressor genes [43] and similar trials to prevent colorectal cancer by inhibiting the enzyme responsible for DNA methylation [44]. Such therapies have shown promise in halting tumor growth by reactivation of the tumor suppressor gene or by blocking progression of precancerous epigenetic lesions.

As a corollary to hypermethylation, the predominance of hypomethylation in our keloid genome-wide scan suggesting activated oncogene transcription rather than tumor suppressor silencing mechanisms in the promotion of tumorigenesis opens up opportunities for drug therapies already mature in oncogenic drug discovery pipelines. This is illustrated for S-adenosylmethionine (SAM), which serves as a major methyl donor in biological transmethylation events. In gastric cancer cells and colon cancer cells with hypomethylated* c-myc* and* h-ras* promoters, treatment with SAM resulted in increased promoter methylation and consequent downregulation of mRNA and protein levels of* c-myc *and* h-ras* [45]. The study found no influence on mRNA and protein levels of p16 (INK4a) with and without SAM treatment, supporting SAM as an effective inhibitor of tumor cell growth by reversing DNA hypomethylation.

This study provides new information about the pathogenesis of keloids highlighting a predominance of hypomethylated genomic landscapes, favoring nonpromoter regions. Utilizing the Infinium HumanMethylation450 BeadChip kit (Illumina, Inc., San Diego, CA), the differentially methylated CpGs indicated 152 keloid-specific promoter region genes, of which 63% (96) are hypomethylated as compared to 37% (56) hypermethylated. Despite a study limitation of a small sample size, the combination of the 450K array with a robust three-tier data analysis approach identified a relatively large number of statistically significant differentially methylated genes. A strength of this study is its strategy as a discovery approach starting from the most comprehensive currently available human methylome platform, providing not only new information of previously unreported genes but also supporting studies of genes already reported in keloids as well as some genes implicated in the pathogenesis of other tumors. For example,* ITGB7 *is a gene that was identified in this pilot study as differentially methylated in keloids compared to normal skin.* ITGB7* is a member of the integrin family which has been found to be upregulated in keloid tissue and keloid fibroblast [46]. Likewise, microRNA genes,* MIR199A2*,* MIR609*, and* MIR938,* were also differentially methylated in this pilot study. These genes are part of a family of short noncoding RNA genes involved in gene regulation. Downregulation of members of MIR7 gene has been implicated in the overproduction of collagen in keloids and local scleroderma [47]. Furthermore, altered expression of* MIR199A* has been implicated in cervical carcinoma and suggests a role in tumorigenesis [48].

Another limitation of the study is the anonymous nature of the sample collection. This prevented stratification based on demographics, treatment, or primary or recurrence status and did not allow for patients to serve as their own control. Matched normal and keloid from the same individuals would have likely allowed for less variation. Robust statistical methods employed in this study were aimed at minimizing these variations. In the context of a pilot study, this comprehensive scan of the keloid methylome was attempted as an initial proof of concept to generate a knowledge base for insight into the biological complexity of keloids from a genome-wide promoter methylation perspective. It should serve as an important step in the further exploration of methylated landscapes, including gene promoter and nonpromoter regions as relevant areas of focus in the pathogenesis and potential treatment of keloids. Keloids are by far the worst scars following injury to the skin. The use of epigenetics to identify a role for methylation in keloids may allow for a better understanding of keloid pathogenesis and could lead us closer to scarless surgery.

## 5. Conclusion

Keloid genomes overall are more hypomethylated than hypermethylated occurring more often in nonpromoter genomic regions. Further unraveling of the regionally methylated genomic landscape in keloids should provide a better understanding of the contribution of the DNA methylome to keloid pathogenesis as an additional mechanism for gene regulation, supporting the contribution of both genetic and epigenetic changes in keloid pathogenesis with the potential for novel and more effective therapeutic strategies.

## Data Access

All normalized and raw data were submitted to GEO (Gene Expression Omnibus, NCBI) according to the instructions provided (GEO accession numbers: GSE56420). For GEO linking and citing, please refer to http://www.ncbi.nlm.nih.gov/geo/info/linking.html.

## Supplementary Material

Supplementary Table 1 lists Tier 3 significantly differentiated CpGs, their corresponding genes, methylation beta values, and status as either hypermethylated or hypomethylated.

## Figures and Tables

**Table 1:** Breakdown of 685 differentially methylated Tier 3 CpGs^1^.

A: methylation status of 685 CpGs	
Hypermethylated	175 (26%)
Hypomethylated	510 (74%)
B: 685-CpG distribution in promoter versus nonpromoter	
Promoter	190 (28%)
Nonpromoter	495 (72%)
C: methylation status of 190 promoter CpGs	
Hypermethylated	62 (33%)
Hypomethylated	128 (67%)
D: 190-CpG distribution in promoter regions	
TSS200	29%
TSS1500	26%
5′UTR	41%
First exon	4%
E: methylation status of 152 genes associated with the 190 CpGs	
Hypermethylated	56 (37%)
Hypomethylated	96 (63%)

^1^CpG (cytosine-phosphodiester bond-guanine); TSS (transcription start site); UTR (untranslated region).
