# Supplementary material for: Genome-Wide Scan for Methylation Profiles in Keloids
Source: Dis Markers. 2015 May 14;2015:943176. doi: 10.1155/2015/943176 (PMC4446486; doi:10.1155/2015/943176)
Supplement: Supplementary file 1 — Supplementary Table 1 lists Tier 3 significantly differentiated CpGs, their corresponding genes, methylation beta values, and status as either hypermethylated or hypomethylated. [file 943176.f1.pdf]

Supplementary Table 1: Tier 3 significantly differentiated CpGs and their corresponding genes

Target ID	Normal (mean β)	Keloid (mean β)	Mean β ratio (keloid vs normal)	Gene Name	Methylation Status
cg21052932	0.6405	0.316151	0.493599	*ABHD12B*	hypomethylated
cg22960869	0.407755	0.130023	0.318876	*ABI3*	hypomethylated
cg01270001	0.056313	0.267278	4.746288	*ACTR3C*	hypemethylated
cg10348193	0.06322	0.283151	4.478798	*ACTR3C*	hypemethylated
cg11026333	0.027539	0.229677	8.339962	*ACTR3C*	hypemethylated
cg21151432	0.124841	0.325219	2.605056	*ADCY3*	hypemethylated
cg12741645	0.414373	0.139782	0.337335	*AEN*	hypomethylated
cg11808936	0.511494	0.234321	0.458112	*AFAP1*	hypomethylated
cg12610832	0.07638	0.327356	4.285899	*AHDC1*	hypomethylated
cg23447569	0.33071	0.67257	2.033719	*AHNAK*	hypemethylated
cg25229172	0.154144	0.354335	2.298722	*AMDHD1*	hypemethylated
cg24552844	0.515027	0.256552	0.498133	*AMICA1*	hypomethylated
cg26547698	0.668441	0.116209	0.173852	*ANKRD11*	hypomethylated
cg25733272	0.202181	0.417417	2.064568	*AOAH*	hypemethylated
cg16066354	0.396425	0.153572	0.387392	*APOBEC3D*	hypomethylated
cg09015973	0.576903	0.262442	0.454915	*ARHGEF4*	hypomethylated
cg22367549	0.410991	0.168195	0.409243	*ATP2B4*	hypomethylated
cg16389901	0.087854	0.35652	4.058102	*BMP4*	hypomethylated
cg15494458	0.405114	0.18764	0.463179	*BPI*	hypomethylated
cg14472181	0.25149	0.519987	2.067625	*BRD3*	hypemethylated
cg11365617	0.415572	0.190492	0.458385	*C12orf34*	hypomethylated
cg24736933	0.119067	0.37742	3.169812	*C15orf52*	hypemethylated
cg04245402	0.316311	0.747164	2.362121	*C19orf21*	hypemethylated
cg13689560	0.347205	0.101869	0.293397	*C19orf36*	hypomethylated
cg24088508	0.050797	0.260099	5.12037	*C1orf109*	hypemethylated
cg04499514	0.581593	0.207134	0.356148	*C3AR1*	hypomethylated
cg09238677	0.355766	0.100977	0.283831	*C3AR1*	hypomethylated
cg12289251	0.231811	0.484501	2.090073	*CACNB2*	hypemethylated
cg25327888	0.067591	0.280677	4.152601	*CACNB2*	hypemethylated
cg06075311	0.32543	0.122811	0.37738	*CALHM2*	hypomethylated

cg24587601	0.566718	0.251257	0.443355	*CASS4*	hypomethylated
cg12154803	0.076925	0.319923	4.158884	*CCDC34*	hypemethylated
cg25229172	0.154144	0.354335	2.298722	*CCDC38*	hypemethylated
cg10439431	0.54123	0.260691	0.481665	*CCL23*	hypomethylated
cg14916288	0.495439	0.217572	0.439149	*CCL23*	hypomethylated
cg11303839	0.556402	0.185663	0.333684	*CCL26*	hypomethylated
cg23817981	0.454894	0.145815	0.320548	*CCR4*	hypomethylated
cg03477080	0.354115	0.122361	0.345539	*CCRL2*	hypomethylated
cg08679238	0.330332	0.120913	0.366033	*CCRL2*	hypomethylated
cg12579212	0.387102	0.136471	0.352546	*CCRL2*	hypomethylated
cg13070763	0.342344	0.116972	0.341679	*CCRL2*	hypomethylated
cg14754581	0.399205	0.157961	0.395689	*CCRL2*	hypomethylated
cg13018120	0.488364	0.195633	0.400589	*CD300E*	hypomethylated
cg23557926	0.316312	0.08085	0.255602	*CFH*	hypomethylated
cg16519762	0.253649	0.521838	2.057327	*CHD3*	hypemethylated
cg01487542	0.589477	0.287697	0.488055	*CMAH*	hypomethylated
cg25832824	0.068498	0.305011	4.452872	*CMKLR1*	hypemethylated
cg18693704	0.207502	0.461046	2.221891	*CMTM4*	hypemethylated
cg06484514	0.1476	0.39528	2.678047	*COL18A1*	hypemethylated
cg23601416	0.264688	0.541629	2.04629	*COMT*	hypemethylated
cg23281552	0.466839	0.213535	0.457406	*CREB5*	hypomethylated
cg03992638	0.547668	0.259675	0.474146	*CXXC5*	hypomethylated
cg18264728	0.536104	0.2584	0.481996	*DAB2*	hypomethylated
cg21037265	0.3711	0.093673	0.252419	*DENND1C*	hypomethylated
cg26597727	0.429102	0.207644	0.483903	*DENND1C*	hypomethylated
cg15354065	0.240065	0.523784	2.181843	*DGKA*	hypemethylated
cg01262913	0.371074	0.142815	0.38487	*DSCR9*	hypomethylated
cg11399508	0.364316	0.145544	0.399498	*DSCR9*	hypomethylated
cg07318313	0.398256	0.167273	0.420013	*ENG*	hypomethylated
cg03316237	0.464197	0.223841	0.482212	*EXOC3L2*	hypomethylated
cg14753355	0.397075	0.141115	0.355386	*F2RL3*	hypomethylated
cg19319487	0.546016	0.234365	0.429227	*FAM168A*	hypomethylated

cg02293775	0.183471	0.386752	2.107974	*FAM53B*	hypemethylated
cg25120326	0.454807	0.226865	0.498817	*FBLN2*	hypomethylated
cg00052684	0.225066	0.497574	2.210792	*FKBP5*	hypemethylated
cg02637352	0.315877	0.663385	2.100135	*FLJ41603*	hypemethylated
cg24208206	0.080884	0.342936	4.239825	*FYCO1*	hypemethylated
cg15307891	0.272924	0.019466	0.071324	*GALNT3*	hypomethylated
cg10517535	0.242268	0.519883	2.145899	*GAS7*	hypemethylated
cg06306927	0.334689	0.085726	0.256137	*GHDC*	hypomethylated
cg20795023	0.435376	0.174743	0.401361	*GIMAP1*	hypomethylated
cg03852144	0.470177	0.200492	0.426419	*GLRX*	hypomethylated
cg10949007	0.433034	0.167379	0.386527	*GLRX*	hypomethylated
cg22689909	0.403821	0.122694	0.303833	*GLRX*	hypomethylated
cg25217276	0.321193	0.112529	0.350346	*GNG4*	hypomethylated
cg22960869	0.407755	0.130023	0.318876	*GNGT2*	hypomethylated
cg26170660	0.43583	0.212553	0.487697	*GPX5*	hypomethylated
cg20664636	0.541633	0.23487	0.433632	*HIC1*	hypomethylated
cg15391590	0.560985	0.233619	0.416444	*ICK*	hypomethylated
cg10262052	0.456882	0.2166	0.474082	*IDO1*	hypomethylated
cg00082235	0.313341	0.111398	0.355516	*IFFO1*	hypomethylated
cg00363813	0.484223	0.173106	0.357492	*IFFO1*	hypomethylated
cg00983904	0.404769	0.100503	0.248297	*IFFO1*	hypomethylated
cg01493517	0.418647	0.170229	0.406618	*IFFO1*	hypomethylated
cg01979888	0.499811	0.179072	0.35828	*IFFO1*	hypomethylated
cg08875705	0.49337	0.122401	0.248092	*IFFO1*	hypomethylated
cg17198308	0.459341	0.158182	0.344367	*IFFO1*	hypomethylated
cg22203219	0.362442	0.1621	0.447245	*IFFO1*	hypomethylated
cg23737737	0.402353	0.105681	0.262658	*IFFO1*	hypomethylated
cg02309273	0.420814	0.16572	0.393807	*INPP5B*	hypomethylated
cg05749969	0.49754	0.205922	0.41388	*INPP5B*	hypomethylated
cg10784030	0.383114	0.159252	0.415677	*INPP5B*	hypomethylated
cg17949727	0.447704	0.123924	0.276799	*INPP5B*	hypomethylated
cg23262274	0.390876	0.190537	0.487463	*INPP5B*	hypomethylated

cg14524975	0.522985	0.214487	0.41012	*ITGB7*	hypomethylated
cg22945413	0.478341	0.229284	0.479332	*JAK1*	hypomethylated
cg11185792	0.485403	0.186616	0.384456	*KCNAB2*	hypomethylated
cg03957481	0.389937	0.151514	0.388561	*KLHDC7B*	hypomethylated
cg04873061	0.265842	0.624071	2.34753	*LAMB3*	hypemethylated
cg03987786	0.38321	0.169782	0.443052	*LOC1001282*	hypomethylated
cg17227257	0.264931	0.616585	2.327342	*LOC1001308*	hypemethylated
cg19255477	0.326651	0.124619	0.381505	*LOC1001321*	hypomethylated
cg26426745	0.399701	0.129105	0.323004	*LOC1001321*	hypomethylated
cg12409149	0.266871	0.609971	2.28564	*LOC1002707*	hypemethylated
cg05429448	0.505679	0.247902	0.490236	*LOC152225*	hypomethylated
cg09550041	0.198849	0.46391	2.332972	*LRRC52*	hypemethylated
cg01270001	0.056313	0.267278	4.746288	*LRRC61*	hypemethylated
cg10348193	0.06322	0.283151	4.478798	*LRRC61*	hypemethylated
cg11026333	0.027539	0.229677	8.339962	*LRRC61*	hypemethylated
cg05433805	0.59619	0.286969	0.481338	*LRRFIP2*	hypomethylated
cg27640763	0.334742	0.134694	0.402381	*LUM*	hypomethylated
cg13702222	0.174473	0.377391	2.163028	*MBNL1*	hypemethylated
cg03078520	0.205279	0.452089	2.202317	*MICAL3*	hypemethylated
cg10769535	0.731889	0.30186	0.41244	*MIR199A2*	hypomethylated
cg03775802	0.530182	0.256064	0.482973	*MIR609*	hypomethylated
cg15856028	0.459369	0.21658	0.471472	*MIR938*	hypomethylated
cg09530163	0.405757	0.129455	0.319044	*MMP2*	hypomethylated
cg13689560	0.347205	0.101869	0.293397	*MOBKL2A*	hypomethylated
cg20761290	0.35095	0.14525	0.413876	*MS4A7*	hypomethylated
cg00121389	0.328109	0.118396	0.360843	*MTUS1*	hypomethylated
cg06752054	0.3473	0.079274	0.228258	*MX2*	hypomethylated
cg21130374	0.517409	0.14933	0.288611	*MX2*	hypomethylated
cg20363347	0.232581	0.46973	2.019635	*MYEOV*	hypemethylated
cg03079497	0.214922	0.432473	2.012233	*MYO1C*	hypemethylated
cg09825309	0.399627	0.188283	0.471147	*NCOR2*	hypomethylated
cg17132902	0.390095	0.178005	0.456313	*NOS1*	hypomethylated

cg10465672	0.122165	0.367512	3.008335	*OCIAD2*	hypemethylated
cg08794954	0.401915	0.175006	0.43543	*ODZ4*	hypomethylated
cg05094216	0.205388	0.439385	2.13929	*P2RY12*	hypemethylated
cg06103394	0.07728	0.437452	5.660611	*PAQR4*	hypemethylated
cg01311181	0.380225	0.149393	0.392907	*PARVG*	hypomethylated
cg05148373	0.299823	0.079243	0.264298	*PDE1A*	hypomethylated
cg08759112	0.502301	0.220188	0.438358	*PDE1A*	hypomethylated
cg10058920	0.361679	0.147459	0.407708	*PDYN*	hypomethylated
cg04142864	0.491347	0.167466	0.340829	*PHLDB1*	hypomethylated
cg20309703	0.357485	0.119581	0.334507	*PHLDB1*	hypomethylated
cg22466012	0.509054	0.143078	0.281067	*PLD1*	hypomethylated
cg22451657	0.356599	0.739616	2.074087	*PLEKHG5*	hypemethylated
cg13645732	0.285474	0.039594	0.138697	*PPP1R13L*	hypomethylated
cg19426388	0.359111	0.149394	0.41601	*PPP1R13L*	hypomethylated
cg02712036	0.372277	0.097159	0.260986	*RAB27A*	hypomethylated
cg01246520	0.25891	0.703227	2.716104	*RAI1*	hypemethylated
cg16656875	0.529236	0.070056	0.132372	*SCML4*	hypomethylated
cg17197774	0.456217	0.217758	0.477312	*SDCBP*	hypomethylated
cg24924577	0.258027	0.560446	2.172047	*SEMA4B*	hypemethylated
cg25310824	0.191992	0.414754	2.160268	*SEPP1*	hypemethylated
cg03052162	0.420995	0.182589	0.433709	*SHMT2*	hypomethylated
cg21982437	0.386161	0.176978	0.458302	*SIGLECP3*	hypomethylated
cg21853021	0.313685	0.081432	0.259597	*SLC22A18*	hypomethylated
cg05090759	0.202769	0.41209	2.032316	*SLC38A11*	hypemethylated
cg01289541	0.448635	0.216168	0.481835	*SLC7A14*	hypomethylated
cg20704148	0.161794	0.768343	4.748892	*SLCO2B1*	hypemethylated
cg01778994	0.467425	0.1999	0.427662	*ST3GAL1*	hypomethylated
cg00464095	0.472693	0.236113	0.499506	*STAR*	hypomethylated
cg07092525	0.354881	0.140659	0.396355	*STAR*	hypomethylated
cg09630404	0.3589	0.146964	0.409486	*STAR*	hypomethylated
cg18406492	0.442985	0.180379	0.40719	*STAR*	hypomethylated
cg14462124	0.632701	0.290959	0.459868	*STK17B*	hypomethylated

cg15856028	0.459369	0.21658	0.471472	*SVIL*	hypomethylated
cg17194270	0.349923	0.709926	2.028806	*SYNGR1*	hypemethylated
cg20496314	0.296262	0.592915	2.001322	*SYNGR1*	hypemethylated
cg02829601	0.422678	0.161128	0.381208	*SYTL3*	hypomethylated
cg09826188	0.111206	0.331275	2.978938	*TBC1D23*	hypemethylated
cg16984944	0.185897	0.392919	2.113641	*TBC1D23*	hypemethylated
cg04272820	0.266479	0.546527	2.050917	*THRA*	hypemethylated
cg22839308	0.498393	0.240268	0.482086	*TLR1*	hypomethylated
cg16585333	0.339926	0.132736	0.390484	*TLR5*	hypomethylated
cg27639199	0.12006	0.658895	5.488059	*TMC3*	hypemethylated
cg15961993	0.633854	0.296372	0.467571	*TMEM108*	hypomethylated
cg00463982	0.381111	0.165119	0.433257	*TMEM204*	hypomethylated
cg06602086	0.501773	0.17139	0.341569	*TMEM204*	hypomethylated
cg07341220	0.405345	0.191743	0.473037	*TMEM204*	hypomethylated
cg15694715	0.274113	0.623154	2.273344	*TMEM25*	hypemethylated
cg14886269	0.114699	0.33983	2.962784	*TNFRSF18*	hypemethylated
cg05802478	0.269599	0.540746	2.005744	*TNK2*	hypemethylated
cg21008828	0.141921	0.370077	2.607617	*TNK2*	hypemethylated
cg21642245	0.125242	0.35061	2.799461	*TNK2*	hypemethylated
cg26740249	0.21973	0.485247	2.208374	*TNRC6C*	hypemethylated
cg09548780	0.145044	0.411983	2.840404	*TNS1*	hypemethylated
cg07241084	0.489432	0.192879	0.394088	*TNS3*	hypomethylated
cg01824466	0.502017	0.237752	0.473593	*TP53INP1*	hypomethylated
cg03270395	0.179376	0.391998	2.185343	*TRAF3IP2*	hypemethylated
cg05423393	0.204691	0.462905	2.261488	*TRAF3IP2*	hypemethylated
cg02735762	0.472618	0.20553	0.434875	*TTF1*	hypomethylated
cg14786652	0.567648	0.27217	0.479469	*TTLL10*	hypomethylated
cg21288685	0.362804	0.161869	0.446162	*TTLL10*	hypomethylated
cg25134306	0.519754	0.234386	0.450956	*UBE2D2*	hypomethylated
cg16719560	0.324209	0.106436	0.328295	*VAMP5*	hypomethylated
cg04814784	0.323919	0.818259	2.526127	*VHL*	hypemethylated
cg09433910	0.497816	0.208153	0.418132	*WDR81*	hypomethylated

cg10080732	0.463315	0.142253	0.307034	*WDR81*	hypomethylated
cg17165848	0.449614	0.105934	0.235612	*WIPF1*	hypomethylated
cg22047295	0.4127	0.088085	0.213436	*WIPF1*	hypomethylated
cg15559674	0.495359	0.23427	0.47293	*ZC3H12D*	hypomethylated
cg04920761	0.100922	0.327336	3.243456	*ZFP36L2*	hypemethylated
cg17280346	0.41156	0.200206	0.486455	*ZIC1*	hypomethylated
cg18121066	0.28358	0.764495	2.695875	*ZNF750*	hypemethylated
